# Decreased selenium-binding protein 1 mRNA expression is associated with poor prognosis in renal cell carcinoma

**DOI:** 10.1186/1477-7819-12-288

**Published:** 2014-09-16

**Authors:** Yun-Sok Ha, Geun Taek Lee, Ye-Hwan Kim, Se Yun Kwon, Seock Hwan Choi, Tae-Hwan Kim, Tae Gyun Kwon, Seok Joong Yun, Isaac Yi Kim, Wun-Jae Kim

**Affiliations:** Department of Urology, School of Medicine, Kyungpook National University Medical Center, Daegu, Korea; Section of Urologic Oncology, Rutgers Cancer Institute of New Jersey, New Brunswick, NJ 08903 USA; Department of Urology, College of Medicine, Chungbuk National University, Cheongju, Korea; Department of Urology, College of Medicine and Institute for Tumor Research, Chungbuk National University, 776 1 Sunhwan-ro, Heungduk-gu, Cheongju, 361-763 Korea

**Keywords:** Renal cell carcinoma, SELEMBP1, Prognostic marker

## Abstract

**Background:**

The anticancer effects of selenium may be mediated by selenium-binding proteins, such as SELENBP1. The association between SELENBP1 expression levels and clinicopathologic parameters was assessed in renal cell carcinoma (RCC).

**Methods:**

SELENBP1 mRNA expression was measured with real-time quantitative polymerase chain reaction (qPCR) in 139 specimens of primary RCC and 59 specimens of donor-matched normal-appearing kidney tissues. The prognostic effect of SELENBP1 levels was evaluated with Kaplan–Meier and multivariate Cox regression analyses.

**Results:**

SELENBP1 mRNA levels were significantly lower in tumor tissues than in matched normal kidney tissues (*P* < 0.001) and significantly inversely correlated with pathologic (T-stage and Fuhrman grade) and prognostic variables (progression and cancer-specific death). Kaplan–Meier estimates showed that low SELENBP1 expression was significantly correlated with cancer-specific death (log-rank test, *P* = 0.014), and a multivariate Cox regression model revealed that SELENBP1 expression was an independent predictor of cancer-specific death (HR, 0.111; *P* = 0.006).

**Conclusions:**

SELENBP1 might play a role in tumor suppression and could be a useful prognostic factor in RCC.

## Background

Kidney cancer composes 5% and 3% of malignancies in male and female patients, respectively, with approximately 63,920 new diagnoses in 2014 and 13,860 deaths of the disease in the United States [1]. Renal cell carcinoma (RCC) is the most frequently occurring malignant tumor of the kidney in adults [2]. Approximately one third of RCC patients experience local or distant recurrence after definitive surgery [3]. Recognized prognostic factors, such as pathologic staging and histologic grading, are not sufficient for prognosis when used alone [4]. Therefore, it is crucial to identify biologic markers to find patients at high risk for disease progression.

Selenium is a trace element that is essential for a number of biologic processes. Supplemental dietary selenium was first observed to play a role in reducing cancer risk more than 40 years ago [5]. A deficiency in dietary selenium is associated with an increased incidence of epithelial cancers, including lung, liver, colorectal, and prostate cancer [6]. The antitumor function of selenium is thought to be mediated through selenium-binding protein 1 (SELENBP1). The SELENBP1 gene is located on chromosome 1q21–22; the mRNA sequence of the gene is composed of 1,721 nucleotides encoding 640 amino acids [7]. SELENBP1 mRNA is abundantly expressed in many types of tissues [8]. Its expression is reduced markedly in multiple epithelial cancers compared with normal tissues, suggesting a possible link to malignancies associated with selenium deficiencies [9–13]. Moreover, reduced selenium-binding protein 1 expression is associated with poor outcome in various human cancers [9–12, 14, 15]. Therefore, SELENBP1 may play a critical role in regulating malignant transformation and cancer progression. Nevertheless, little information is available on the expression and function of SELENBP1 during the RCC carcinogenic process in humans, and the significance of SELENBP1 expression in RCC is still largely unknown.

The aims of the present study were (a) to compare the expression level of *SELENBP1* in the tumor with that in normal adjacent tissue, and (b) to define the value of *SELENBP1* expression for predicting tumor outcomes, such as progression and cancer-related death.

## Methods

### Study population and clinical specimens

Between April 1996 and December 2010, RCC samples were obtained from 139 patients with primary RCC who underwent radical nephrectomy or partial nephrectomy at the Chungbuk National University Hospital. Donor-matched normal-appearing kidney tissues (≥5 mm from the tumor tissue) were obtained from 59 patients. The study was in agreement with the Declaration of Helsinki and received Institutional Review Board approval (IRB approval number 2006-01-001). All participating patients gave written informed consent. All tumors were macrodissected within 15 minutes of surgical resection, flash-frozen in liquid nitrogen, and stored at −80°C until use. Staging of RCC was performed as per the American Joint Committee on Cancer (AJCC) staging manual [16]. Histologic differentiation was evaluated by using the Fuhrman nuclear grading system [17]. All patients were evaluated postoperatively every 3 months for the first 2 years, every 6 months for the following 2 years, and yearly thereafter. The definition of disease progression included local recurrence, lymph node metastasis, and distant metastasis by CT scan and bone scan.

### RNA extraction and construction of cDNA

Total RNA was separated from tissue homogenized in a 5-ml glass tube in 1 ml TRIzol (Invitrogen, Carlsbad, CA, USA). The homogenate was transferred to a 1.5-ml tube and mixed with 200 μl of chloroform. After incubation for 5 minutes at 4°C, the homogenate was centrifuged for 13 minutes at 13,000 *g* and 4°C. The upper aqueous phase was transferred to a clean tube, 500 μl of isopropanol was added, and the mixture was incubated for 60 minutes at 4°C. The sample was then centrifuged for 8 minutes at 13,000 *g* and 4°C. Then the upper aqueous phase was removed, mixed with 500 μl of 75% ethanol, and centrifuged for 5 minutes at 13,000 *g* and 4°C. After the upper aqueous layer was discarded, the pellet was dried at room temperature, dissolved with diethylpyrocarbonate (DEPC)-treated water, and stored at −80°C. The quality and integrity of the RNA were confirmed with agarose gel electrophoresis and ethidium bromide staining. cDNA was then prepared from 1 μg of total RNA by using the First-Strand cDNA Synthesis kit (Clontech, TAKARA, Otsu, Japan) according to the manufacturer’s protocol.

### Real-time quantitative polymerase chain reaction (qPCR)

To quantify mRNA expression levels, qPCR amplification was performed by using a Rotor-Gene 6000 instrument (Corbett Research, Mortlake, Australia). qPCR assays were carried out in microreaction tubes (Corbett Research) by using SYBR premix EX Taq (TAKARA BIO Inc., Otsu, Japan) and *SELENBP1* primers. The PCR reaction was performed in a final volume of 10 μl, consisting of 5 μl of 2 × SYBR premix EX Taq buffer, 0.5 μl of each primer (10 p*M*/μl), and 2 μl of cDNA. The product was purified with a QIAquick Extraction kit (QIAGEN, Hilden, Germany), quantified with a spectrometer (Perkin Elmer MBA-2000, Fremont, CA, USA), and sequenced with an automated laser fluorescence sequencer (ABI PRISM 3100 Genetic Analyzer, Foster City, CA, USA). The product was serially diluted from 100 pg/μl to 0.1 pg/μl to establish a standard curve. The qPCR conditions were 1 cycle at 96°C for 20 seconds, followed by 40 cycles of 3 seconds at 96°C for denaturation, 15 seconds at 60°C for annealing, and 15 seconds at 72°C for extension. The melting program was performed at 72°C to 95°C with a heating rate of 1°C per 45 seconds. Spectral data were captured and analyzed by using the Rotor-Gene Real-Time Analysis Software 6.0 Build 14 (Corbett Research). All samples were run in triplicate. *Glyceraldehyde-3-phosphate dehydrogenase* (*GAPDH*) was used as a reference gene. *SELENBP1* expression was normalized to that of *GAPDH.* Primer sequences are shown in Table 1.

**Table 1:** **PCR primer sequences**

Human SELENBP1 forward	GGGAGGTACATGGTCAGTGG
Human SELENBP1 reverse	GGAAGAGCTGTCCTGTGAGG
Human GAPDH forward	TGCACCACCAACTGCTTAGC
Human GAPDH reverse	GGCATGGACTGTGGTCATGAG

### Statistics

To normalize the highly skewed distribution of SELENBP1 mRNA expression, the data were examined as natural log and subsequently back-transformed to express the model results as geometric mean (antilog 95% confidence interval (CI) [18]. To compare gene-expression levels among the groups, a two-sample *t* test or ANOVA was performed. Spearman correlation coefficients were performed to evaluate the association between the SELENBP1 expression and clinicopathologic parameters. Patients were classified as having high or low expression of SELENBP1, with the median expression (0.53 × 10^6^ copies/μl) as the cutoff value. The Kaplan–Meier method was used to estimate the time to progression and cancer-specific death, and differences were assessed by using log-rank statistics. The prognostic value of SELENBP1 expression was analyzed by using a multivariate Cox proportional hazards regression model. Statistical analysis was performed by using IBM SPSS ver. 20.0 (IBM Co., Armonk, NY, USA), and *P* < 0.05 was considered statistically significant.

## Results

### SELENBP1 mRNA expression in RCC and surrounding donor-matched normal tissues

**Figure 1:**
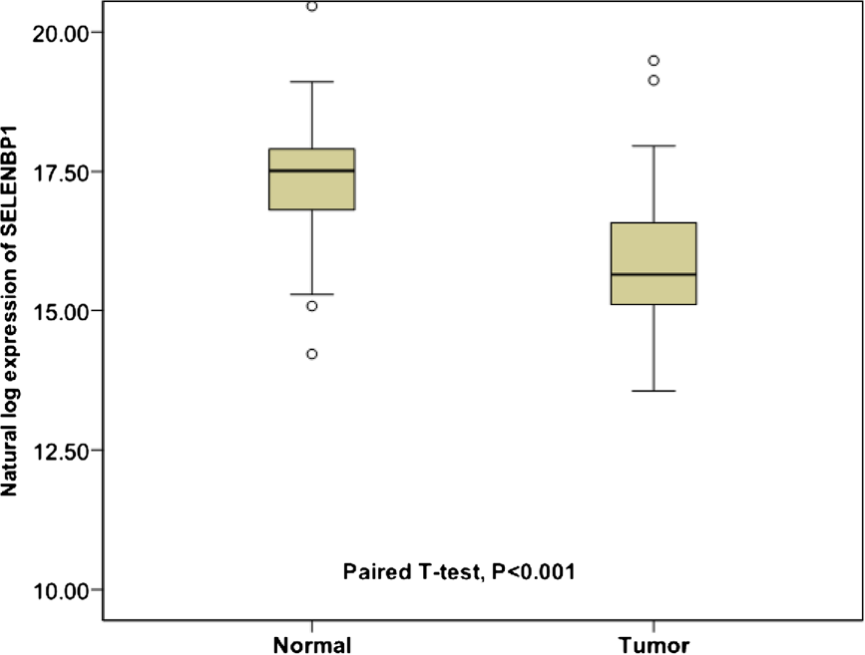
**Selenium**-**binding protein 1 (SELENBP1) mRNA levels in tumor tissues and adjacent normal tissues in 59 patients with renal cell carcinoma (RCC) assessed with qPCR.**

Paired tissue samples (tumor tissue and donor-matched adjacent normal tissue) were collected from 59 patients with RCC. As shown in Figure 1, the mRNA level of SELENBP1 in RCC tissues was apparently lower than in normal adjacent kidney tissues (*P* < 0.001).

### Baseline characteristics in RCC patients

Table 2 lists the baseline characteristics of the 139 RCC patients recruited in this study. The median age of the RCC patients was 58 years (range, 21 to 83 years). At the time of diagnosis, 114 (82%) patients had local disease (pT1/pT2), and 25 (18%) had locally advanced disease (pT3/pT4). The Fuhrman nuclear grades were 1, 2, 3, and 4 in 25 (18.0%), 58 (41.7%), 43 (30.7%), and 13 (9.4%) cases, respectively. Ten (7.2%) N^+^ and nine (6.5%) M^+^ patients were in our study population. The median follow-up period was 42.9 months (range, 1.0 to 156.8 months).

**Table 2:** **Baseline characteristics**

Variables	Incidence (%) or value
Median age (range)	58 years (21–83)
Median follow-up periods (range)	42.9 months (1.0–156.8)
Gender	
Male	103 (74.1)
Female	36 (25.9)
Histologic subtype	
Conventional	117 (84.2)
Papillary	16 (11.5)
Chromophobe	5 (3.6)
Unclassified	1 (0.7)
Pathologic T stage	
pT1a	59 (42.4)
pT1b	34 (24.5)
pT2	21 (15.1)
pT3	20 (14.4)
pT4	5 (3.6)
N-stage	
N0 or Nx	129 (92.8)
N^+^	10 (7.2)
M stage	
M0 or Mx	130 (93.5)
M1	9 (6.5)
Nuclear grade	
1	25 (18.0)
2	58 (41.7)
3	43 (30.7)
4	13 (9.4)

### Relation between SELENBP1 mRNA expression levels and clinicopathologic features

The expression of SELENBP1 mRNA was significantly lower in cancer specimens from patients with high-grade, locally advanced T stage (pT3/pT4), progression, and cancer-specific death than in those with low-grade, local disease (pT1/pT2) and nonprogression who survived or died of other causes than RCC (*P* = 0.042, *P* = 0.034, *P* = 0.009, and *P* = 0.002, respectively) (Table 3). On correlation analysis, the SELENBP1 expression correlated significantly with pathologic stage (*r = −*0.200; *P =* 0.018). However, no significant correlation was found between Fuhrman grade and SELENBP1 expression (*r = −*0.072; *P =*0.399). When we examined the tumor diameters, the median tumor size was 4.5 cm (range, 1–17 cm). SELENBP1 mRNA levels showed significant correlation with tumor dimensions (*r = −*0.184; *P =* 0.030).

**Table 3:** **mRNA expression of SELENBP1 versus clinicopathologic parameters in RCC**

Parameters ( *N*)	mRNA expression level (×10 ^6^ copies/μl)	*P*
Pathologic T-stage (Low versus High stage)		0.034
pT1–2 (114)	0.64 (0.51–0.81)	
pT3–4 (25)	0.36 (0.22–0.57)	
T stage (individual stage)		0.030
pT1 (93)	0.64 (0.50–0.82)	
pT2 (21)	0.55 (0.35–0.70)	
pT3 (20)	0.40 (0.23–0.61)	
pT4 (5)	0.23 (0.10–0.41)	
N stage		0.267
N0 or Nx (129)	0.60 (0.48–0.74)	
N^+^ (10)	0.38 (0.16–0.86)	
M stage		0.855
M0 or Mx	0.58 (0.47–0.72)	
M1	0.54 (0.24–1.21)	
Nuclear grade		
1 (25)	0.63 (0.43–0.94)	0.042
2 (58)	0.59 (0.42–0.83)	
3 (43)	0.70 (0.48–1.03)	
4 (13)	0.23 (0.11–0.49)	
Progression		0.009
No (115)	0.65 (0.52–0.82)	
Yes (24)	0.31 (0.19–0.52)	
Cancer-specific death		0.002
No (122)	0.65 (0.52–0.81)	
Yes (17)	0.24 (0.13–0.421)	

### Prognostic value of SELENBP1 mRNA expression levels for progression and cancer-specific death in RCC

**Figure 2:**
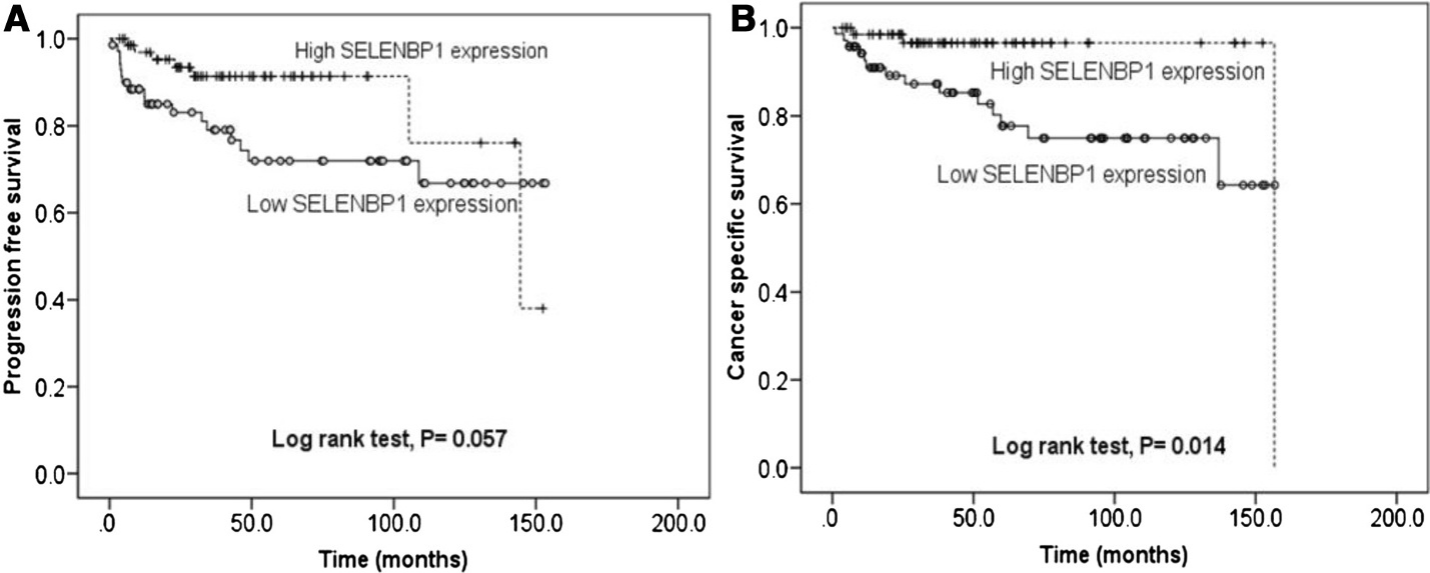
**Effect of SELENBP1 expression on progression**-**free survival (A) and cancer**-**specific survival (B).**

Kaplan–Meier analysis revealed prolonged cancer-specific survival in high-SELENBP1 expressors compared with low expressors in RCC (*P* = 0.014) (Figure 2B). Progression-free survival had marginal association with SELENBP1 (*P* = 0.059) (Figure 2A). Univariate analysis using a Cox proportional hazards model to evaluate the potential utility of SELENBP1 mRNA expression as a prognostic marker in RCC after surgery showed that SELENBP1 expression (*P* = 0.019), Fuhrman grade (*P* = 0.010), N stage (*P* < 0.001), M stage (*P* < 0.001), and T stage (*P* < 0.001) were prime variables for cancer-related death (Table 4). After adjusting for clinicopathologic variables, SELENBP1 expression (*P* = 0.006), N stage (*P* < 0.001), and T stage (*P* = 0.033) remained significantly correlated with cancer-related death in RCC (Table 4).

**Table 4:** **Univariate and multivariate Cox regression analysis for prediction of cancer**-**related death in RCC**

Variables	Univariate		Multivariate	
	HR (95% CI)	*P*	HR (95% CI)	*P*
Age (<58 versus ≥ 58)	1.372 (0.510–3.692)	0.531	1.249 (0.427–3.651)	0.685
Sex (male versus female)	0.984 (0.343–2.822)	0.976	0.953 (0.260–4.615)	0.532
T stage (T1–2 versus T3–4)	9.378 (3.424–25.689)	<0.001	4.432 (1.127–17.425)	0.033
N Stage (N0 or Nx versus N^+^)	15.602 (5.304–45.892)	<0.001	20.373 (4.439–93.498)	<0.001
M Stage (M0 or Mx versus M1)	8.264 (2.826–24.166)	<0.001	1.761 (0.529–5.859)	0.356
Fuhrman grade (1–2 versus 3–4)	3.996 (1.402–11.392)	0.010	3.015 (0.697–13.033)	0.140
SELENBP expression (low versus high)	0.168 (0.038–0.741)	0.019	0.111 (0.023–0.529)	0.006

## Discussion

Biologic markers may enhance our understanding of the pathogenesis of RCC and have value in prognosis and treatment. Lucas *et al*. [19] showed that the downregulation of hepatocyte nuclear factor 4 alpha (HNF4α) in RCC could be interpreted as an indication that (HNF4α) plays a role as a tumor suppressor. Bui *et al.* [20] reported that Ki67 and carbonic anhydrase IX (CA9) were significant predictors of survival and histologic grade. Hypoxia-induced factor-1 alpha (HIF-1a) was found to be an independent prognostic factor for patients with metastatic clear cell RCC [21]. Jacobsen *et al*. [22] found that serum vascular endothelial growth factor (VEGF) level was significantly correlated to tumor stage and grade. Serum C-reactive protein (CRP) was also a robust predictor of metastasis and mortality after curative nephrectomy [23, 24]. From the report from Lucarelli *et al.* [25], a famous tumor marker CA 15–3 was an independent adverse predictor for cancer-specific survival in RCC patients [25]. Despite intensive effort, no biologic markers are available for routine use in the clinic.

SELENBP1, a member of the selenoprotein family, binds selenium covalently and mediates the intracellular transport of selenium [26–28]. Epidemiologic and clinical trial data demonstrate that a deficiency in dietary selenium is associated with an increased incidence of epithelial cancers [6, 29]. Nutritional levels of selenoproteins are enough to mediate the anticancer properties of selenium. Little information is available on the expression and function of SELENBP1 during the RCC carcinogenic process in humans. The present results suggest for the first time that downregulation of SELENBP1 may be involved in human RCC tumorigenesis and be an independent predictor of cancer-specific death in RCC.

SELENBP1 is downregulated in several tumor types. In a study of breast cancer samples, the level of SELENBP1 was decreased in tumor tissues compared with normal tissues and correlated with late disease stages and poor survival [30]. In addition, Zeng *et al*. [31] detected progressive reduction of SELENBP1 during the human bronchial epithelial carcinogenic process and found that the expression levels of SELENBP1 could distinguish normal bronchial epithelium from preneoplastic lesions and invasive lung squamous cell cancer. Similarly, reduced levels of SELENBP1 are an indicator of poor prognosis in colon cancer [10, 14]. In prostate cancer, low levels of SELENBP1 were suggestively associated with increased Gleason Score and poor clinical outcome [32]. Zhang *et al.* [33] demonstrated a significant decrease in SELENBP1 in uterine leiomyoma compared with normal myometrium, suggesting that SELENBP1 may be performing its normal biologic function in healthy myometrium. Similar results were obtained in gastric cancer, in which SELENBP1 was detected in all cases of nonneoplastic epithelial tissues but was absent in gastric cancer [34]. These findings indicate that suppression of SELENBP1 might be a late molecular event in gastric carcinoma. The pattern of SELENBP1 staining may provide useful information about the molecular changes that occur during gastric carcinogenesis. Our results in RCC are consistent with these previous findings.

Our study is not without weaknesses. First, the numbers of enrolled samples were relatively small, and the study design was retrospective. Second, the exact mechanisms by which SELENBP1 contributes to tumorigenesis in RCC are not known. *In vitro* and *in vivo* laboratory research is needed to elucidate these mechanisms. Third, we did not evaluate the protein level of SELENBP1, such as by Western blot or immunohistochemical (IHC) staining. In particular, IHC staining of SELENBP1 in RCC tissues is planned. Prospective studies in larger patient populations with longer follow-up periods will improve our understanding of the value of SELENBP1 in RCC prognosis.

## Conclusions

In conclusion, our study found that reduced SELENBP1 mRNA expression might play an important role in RCC tumorigenesis: low SELENBP1 mRNA expression correlates with aggressive disease and predicts cancer-specific survival in RCC.

## Abbreviations

CI: Confidence interval
RCC: renal cell carcinoma
qPCR: quantitative polymerase chain reaction
SELENBP1: selenium-binding protein 1.

## References

[1] Siegel R, Ma J, Zou Z, Jemal A (2014). Cancer statistics, 2014. CA Cancer J Clin.

[2] Mazzucchelli R, Galosi AB, Scarpelli M, Lopez-Beltran A, Cheng L, Montironi R (2014). Contemporary update on pathology-related issues of adult renal neoplasms. Anal Quant Cytopathol Histpathol.

[3] Pantuck AJ, Zisman A, Belldegrun AS (2001). The changing natural history of renal cell carcinoma. J Urol.

[4] Amin MB, Tamboli P, Javidan J, Stricker H, de-Peralta Venturina M, Deshpande A, Menon M (2002). Prognostic impact of histologic subtyping of adult renal epithelial neoplasms: an experience of 405 cases. Am J Surg Pathol.

[5] Shamberger RJ, Frost DV (1969). Possible protective effect of selenium against human cancer. Can Med Assoc J.

[6] Virtamo J, Valkeila E, Alfthan G, Punsar S, Huttunen JK, Karvonen MJ (1987). Serum selenium and risk of cancer: a prospective follow-up of nine years. Cancer.

[7] Chang PW, Tsui SK, Liew C, Lee CC, Waye MM, Fung KP (1997). Isolation, characterization, and chromosomal mapping of a novel cDNA clone encoding human selenium binding protein. J Cell Biochem.

[8] Lanfear J, Fleming J, Walker M, Harrison P (1993). Different patterns of regulation of the genes encoding the closely related 56 kDa selenium- and acetaminophen-binding proteins in normal tissues and during carcinogenesis. Carcinogenesis.

[9] Chen G, Wang H, Miller CT, Thomas DG, Gharib TG, Misek DE, Giordano TJ, Orringer MB, Hanash SM, Beer DG (2004). Reduced selenium-binding protein 1 expression is associated with poor outcome in lung adenocarcinomas. J Pathol.

[10] Li T, Yang W, Li M, Byun DS, Tong C, Nasser S, Zhuang M, Arango D, Mariadason JM, Augenlicht LH (2008). Expression of selenium-binding protein 1 characterizes intestinal cell maturation and predicts survival for patients with colorectal cancer. Mol Nutr Food Res.

[11] Huang KC, Park DC, Ng SK, Lee JY, Ni X, Ng WC, Bandera CA, Welch WR, Berkowitz RS, Mok SC, Ng SW (2006). Selenium binding protein 1 in ovarian cancer. Int J Cancer.

[12] Yang M, Sytkowski AJ (1998). Differential expression and androgen regulation of the human selenium-binding protein gene hSP56 in prostate cancer cells. Cancer Res.

[13] Li LS, Kim H, Rhee H, Kim SH, Shin DH, Chung KY, Park KS, Paik YK, Chang J (2004). Proteomic analysis distinguishes basaloid carcinoma as a distinct subtype of nonsmall cell lung carcinoma. Proteomics.

[14] Kim H, Kang HJ, You KT, Kim SH, Lee KY, Kim TI, Kim C, Song SY, Kim HJ, Lee C (2006). Suppression of human selenium-binding protein 1 is a late event in colorectal carcinogenesis and is associated with poor survival. Proteomics.

[15] Zhang J, Dong WG, Lin J (2011). Reduced selenium-binding protein 1 is associated with poor survival rate in gastric carcinoma. Med Oncol.

[16] Greene FL (2002). The American Joint Committee on Cancer: updating the strategies in cancer staging. Bull Am Coll Surg.

[17] Fuhrman SA, Lasky LC, Limas C (1982). Prognostic significance of morphologic parameters in renal cell carcinoma. Am J Surg Pathol.

[18] Bland JM, Altman DG (1996). Transformations, means, and confidence intervals. BMJ.

[19] Lucas B, Grigo K, Erdmann S, Lausen J, Klein-Hitpass L, Ryffel GU (2005). HNF4alpha reduces proliferation of kidney cells and affects genes deregulated in renal cell carcinoma. Oncogene.

[20] Bui MH, Visapaa H, Seligson D, Kim H, Han KR, Huang Y, Horvath S, Stanbridge EJ, Palotie A, Figlin RA, Belldegrun AS (2004). Prognostic value of carbonic anhydrase IX and KI67 as predictors of survival for renal clear cell carcinoma. J Urol.

[21] Klatte T, Seligson DB, Riggs SB, Leppert JT, Berkman MK, Kleid MD, Yu H, Kabbinavar FF, Pantuck AJ, Belldegrun AS (2007). Hypoxia-inducible factor 1 alpha in clear cell renal cell carcinoma. Clin Cancer Res.

[22] Jacobsen J, Rasmuson T, Grankvist K, Ljungberg B (2000). Vascular endothelial growth factor as prognostic factor in renal cell carcinoma. J Urol.

[23] Johnson TV, Abbasi A, Owen-Smith A, Young A, Ogan K, Pattaras J, Nieh P, Marshall FF, Master VA (2010). Absolute preoperative C-reactive protein predicts metastasis and mortality in the first year following potentially curative nephrectomy for clear cell renal cell carcinoma. J Urol.

[24] de Martino M, Klatte T, Seemann C, Waldert M, Haitel A, Schatzl G, Remzi M, Weibl P (2013). Validation of serum C-reactive protein (CRP) as an independent prognostic factor for disease-free survival in patients with localised renal cell carcinoma (RCC). BJU Int.

[25] Lucarelli G, Ditonno P, Bettocchi C, Vavallo A, Rutigliano M, Galleggiante V, Larocca AM, Castellano G, Gesualdo L, Grandaliano G, Selvaggi FP, Battaglia M (2014). Diagnostic and prognostic role of preoperative circulating CA 15–3, CA 125, and beta-2 microglobulin in renal cell carcinoma. Dis Markers.

[26] Behne D, Kyriakopoulos A (2001). Mammalian selenium-containing proteins. Annu Rev Nutr.

[27] Jeong JY, Wang Y, Sytkowski AJ (2009). Human selenium binding protein-1 (hSP56) interacts with VDU1 in a selenium-dependent manner. Biochem Biophys Res Commun.

[28] Porat A, Sagiv Y, Elazar Z (2000). A 56-kDa selenium-binding protein participates in intra-Golgi protein transport. J Biol Chem.

[29] Klein EA (2004). Selenium and vitamin E cancer prevention trial. Ann N Y Acad Sci.

[30] Zhang S, Li F, Younes M, Liu H, Chen C, Yao Q (2013). Reduced selenium-binding protein 1 in breast cancer correlates with poor survival and resistance to the anti-proliferative effects of selenium. PLoS One.

[31] Zeng GQ, Yi H, Zhang PF, Li XH, Hu R, Li MY, Li C, Qu JQ, Deng X, Xiao ZQ (2013). The function and significance of SELENBP1 downregulation in human bronchial epithelial carcinogenic process. PLoS One.

[32] Jerome-Morais A, Wright ME, Liu R, Yang W, Jackson MI, Combs GF, Diamond AM (2012). Inverse association between glutathione peroxidase activity and both selenium-binding protein 1 levels and Gleason score in human prostate tissue. Prostate.

[33] Zhang P, Zhang C, Wang X, Liu F, Sung CJ, Quddus MR, Lawrence WD (2010). The expression of selenium-binding protein 1 is decreased in uterine leiomyoma. Diagn Pathol.

[34] Zhang J, Zhan N, Dong WG (2011). Altered expression of selenium-binding protein 1 in gastric carcinoma and precursor lesions. Med Oncol.

